# A calmodulin‐like protein regulates plasmodesmal closure during bacterial immune responses

**DOI:** 10.1111/nph.14599

**Published:** 2017-05-17

**Authors:** Bo Xu, Cécilia Cheval, Anuphon Laohavisit, Bradleigh Hocking, David Chiasson, Tjelvar S. G. Olsson, Ken Shirasu, Christine Faulkner, Matthew Gilliham

**Affiliations:** ^1^ Australian Research Council Centre of Excellence in Plant Energy BiologyWaite Research InstituteUniversity of AdelaideGlen OsmondSA5064Australia; ^2^ School of Agriculture, Food and WineWaite Research InstituteUniversity of AdelaideGlen OsmondSA5064Australia; ^3^ John Innes CentreNorwich Research ParkColney LaneNorwichNR4 7UHUK; ^4^ RIKEN Centre for Sustainable Resource ScienceTsurumi‐kuYokohama230‐0045Japan

**Keywords:** At3g50770, biotic stress, cell‐to‐cell communication, electrophoresis mobility shift, maltose‐binding protein, pathogen‐associated molecular pattern (PAMP)

## Abstract

- Plants sense microbial signatures via activation of pattern recognition receptors (PPRs), which trigger a range of cellular defences. One response is the closure of plasmodesmata, which reduces symplastic connectivity and the capacity for direct molecular exchange between host cells.
- Plasmodesmal flux is regulated by a variety of environmental cues but the downstream signalling pathways are poorly defined, especially the way in which calcium regulates plasmodesmal closure.
- Here, we identify that closure of plasmodesmata in response to bacterial flagellin, but not fungal chitin, is mediated by a plasmodesmal‐localized Ca^2+^‐binding protein Calmodulin‐like 41 (CML41). *CML41* is transcriptionally upregulated by flg22 and facilitates rapid callose deposition at plasmodesmata following flg22 treatment. CML41 acts independently of other defence responses triggered by flg22 perception and reduces bacterial infection.
- We propose that CML41 enables Ca^2+^‐signalling specificity during bacterial pathogen attack and is required for a complete defence response against *Pseudomonas syringae*.

## Introduction

Plasmodesmata are plasma membrane‐lined pores that connect the cytoplasm of adjoining plant cells, allowing the passage of small molecules and ions through the plant symplast (Lucas & Lee, 2004). Our understanding of plasmodesmata has grown in the last decade (Lucas & Lee, 2004; Lee & Lu, 2011; Han & Kim, 2016; Tilsner *et al*., 2016). For instance, it is now clear that plasmodesmata dynamically regulate cell‐to‐cell connectivity during developmental transitions and in response to environmental change, e.g. the down‐regulation of plasmodesmal flux is an essential defence response following pathogen attack (Lee *et al*., 2011; Faulkner *et al*., 2013).

Bacterial flagellin triggers many defence responses via the perception of the immunogenic peptide of flagellin (flg22) by its cognate receptor FLS2 (Gómez‐Gómez & Boller, 2000). In Arabidopsis these responses include both the influx of Ca^2+^ and plasmodesmal closure; Ca^2+^ signalling is believed to be a critical component of a successful immune response (Lecourieux *et al*., 2006; Seybold *et al*., 2014). Previous studies have shown that changes in cytosolic Ca^2+^ concentration close plasmodesmata (Tucker & Boss, 1996; Holdaway‐Clarke *et al*., 2000) – this has led to speculation that Ca^2+^ signals regulate plasmodesmal flux following pathogen perception (e.g. Han & Kim, 2016; Tilsner *et al*., 2016). While plasmodesmata‐located calcium responsive proteins and putative calmodulin‐binding sites have been identified (Baluška *et al*., 1999; Chen *et al*., 2005; Fernandez‐Calvino *et al*., 2011; Vaddepalli *et al*., 2014) none have been characterized for their specific role in plasmodesmal function.

Here, we identify Calmodulin‐like protein 41 (CML41) as a plasmodesmata‐located, Ca^2+^ responsive protein that mediates flg22‐induced callose‐dependent plasmodesmal closure. *CML41* expression is upregulated by flg22 and positively regulates defence against *Pseudomonas syringae*; therefore, we have identified a novel component of plant defence that links Ca^2+^ signals with callose deposition and plasmodesmata closure.

## Materials and Methods

### Plant material and growth conditions

*Arabidopsis thaliana* ecotype Col‐0 and transgenic plants were grown in soil under short day conditions (9 h : 15 h, light : dark, 22°C) for 5–6 wk (Conn *et al*., 2013), unless indicated otherwise.

### Gene cloning and plasmid construction

The coding sequence of *CML41* (At3g50770) with or without a stop codon was cloned via PCR (Phusion™ Hot Start High‐Fidelity DNA polymerase; Finnzymes, Vantaa, Finland) with the primers listed in Supporting Information Table S1. To silence *CML41*, an artificial micro RNA (amiRNA, 5′‐TAAACCGTCATCATTTGACCA‐3′) was designed against the *CML41* mRNA sequence using Web Micro RNA Designer (Wmd3, http://wmd3.weigelworld.org/cgi-bin/webapp.cgi) (Schwab *et al*., 2006). Whilst a 2‐kb sequence upstream from the *CML41* ATG start codon was amplified by PCR to represent the *CML41* promoter (*proCML41*). All these PCR products were cloned via the Gateway^®^ system (Invitrogen, Thermo Fisher Scientific, Waltham, MA, USA) following the manufacturers’ instructions. The *CML41* gene with a stop codon was recombined into pDEST566 for protein expression in *Escherichia coli* and into the binary vector pMDC32 for plant overexpression. *CML41* without the stop codon was recombined into the binary vector pMDC83 containing a green fluorescent protein (GFP) tag on the C‐terminus for protein localization and *CML41‐amiRNA* was recombined into the binary vector pMDC32 for knockdown of *CML41* in the plant. The *proCML41* was recombined into the binary vector pMDC162 for a GUS histochemical assay (Curtis & Grossniklaus, 2003) and *35S:PDLP1‐mCherry* was made by Golden Gate cloning. Stable transformation of Arabidopsis was performed by floral dip and T3 homozygote plants were used for all experiments.

### Subcellular localization

Ectopically expressed fluorescent proteins in transgenic Arabidopsis plants were imaged using a confocal laser scanning microscope equipped with a Zeiss Axioskop 2 mot plus LSM5 PASCAL and argon laser (Carl Zeiss, Oberkochen, Germany) or a Leica SP5 confocal microscope (Leica Microsystems, Wexlar, Germany). Sequential scanning and laser excitation was used to capture fluorescence from GFP (excitation = 488 nm, emission = 505–530 nm), aniline blue (excitation = 405 nm, emission = 440–490 nm) and mCherry (excitation = 561 nm, emission = 600–640 nm).

### GUS histochemical analysis

Transgenic *proCML41*::*GUS* plants were stained in the dark using a buffer containing 50 mM sodium phosphate pH = 7.0, 10 mM EDTA, 2 mM potassium ferrocyanide, 2 mM potassium ferricyanide, 0.1% (v/v) Triton X‐100, 0.1% (w/v) X‐Gluc (5‐bromo‐4‐chloro‐3‐indolyl β‐d‐glucuronide) vacuum infiltrated for 15 min, followed by a 3 h incubation at 37°C. The plants were cleared of chlorophyll in 70% ethanol and imaged using a SMZ800 Stereo Fluorescence microscope (Nikon, Tokyo, Japan).

### Quantitative RT‐PCR analysis

RNA was extracted from leaves using TRIzol (Invitrogen) and treated with Turbo DNA‐free kit (Ambion, Thermo Fisher Scientific) before cDNA synthesis using SuperScript^®^ III Reverse Transcriptase (Invitrogen). Quantitative reverse transcription polymerase chain reaction (RT‐PCR) was performed on the cDNA samples with primers listed in Table S1 using the fluorescence output from a QuantStudio™ 12K Flex Real‐Time PCR System. Quantitative RT‐PCR analysis via the 2^−Δ*C*т^ method to calculate the gene expression level relative to either *GAPDH‐A* (At3g26650) or *UBI10* (At4g05320) as an internal control (Schmittgen & Livak, 2008).

### Electrophoresis mobility shift assays

Recombinant *CML41* was expressed in T7 Expression *lysY/I* ^*q*^ *E. coli* cells (New England Biolabs, Ipswich, MA, USA) using a pDEST566 vector tagged with a 6×His maltose‐binding protein (MBP) to enhance solubility of CML41 (Kapust & Waugh, 1999). The recombinant CML41 was purified using Poly‐Prep^®^ Chromatography Columns (Bio‐Rad, Hercules, CA, USA) and Talon^®^ Metal Affinity Resin (Clontech, Mountain View, CA, USA) and desalted using Zeba™ Spin Desalting Columns (Thermo Fisher Scientific) following the manufacturers’ guide. The electrophoresis mobility shift assay was optimized from methods previously described (Garrigos *et al*., 1991). Either 1 mM CaCl_2_ or 10 mM EGTA (ethylene glycol‐bis(β‐aminoethyl ether)‐*N*,*N*,*N*′,*N*′‐tetraacetic acid) was added to the purified desalted recombinant protein samples. These samples were heated at 95°C for 2 min before electrophoresis separation on an 8% SDS‐PAGE gel containing either 1 mM CaCl_2_ or 10 mM EGTA. The mobility of proteins was determined by comparison with the Precision Plus Protein™ Standards (Bio Rad).

### Plasmodesmal callose staining assay

The eighth rosette leaf was infiltrated with ultrapure water, or ultrapure water containing 100 nM flg22 or 1 mM EGTA for 30 min, followed by an infiltration of aniline blue (0.01% (w/v) in PBS buffer, pH 7.4). Callose deposits were imaged from the abaxial side of the leaf using a SP5 confocal microscope (Leica) or a Nikon A1R laser scanning confocal with excitation = 405 nm and emission = 500–550 nm. Z‐stack images were collected from 24 to 27 technical replicates from two independent biological replicates. Data from different microscopes was not pooled. Callose was quantified using automated image analysis. All annotated images were inspected before inclusion of any data in the statistical analysis. The image analysis pipeline was written in Python; image analysis scripts and further information are available under the open source MIT licence on GitHub (https://github.com/JIC-CSB/find-plasmodesmata).

### Macroscopic callose deposition assay

Either ultrapure H_2_O or ultrapure H_2_O containing 1 μM flg22 was infiltrated into rosette leaves. After 24 h, the infiltrated leaves were incubated in staining solution (150 mM sodium phosphate, 0.05% (w/v) aniline blue, pH = 8) for an additional 1 h in the dark. The stained leaves were mounted in 50% (v/v) glycerol and imaged with an Axiophot Photomicroscope excitation from a mercury light source and captured with a UV filter (LP = 470 nm) (Carl Zeiss). Callose deposited in leaves was measured by ImageJ, using particle analysis (http://rsbweb.nih.gov/ij/).

### Reactive oxygen species (ROS) burst assay

Leaf discs were obtained by using a 4 mm disposable biopsy punch (Kei Medical, Tokyo, Japan) and incubated overnight with 100 μM L‐012 (Wako Chemical, Osaka, Japan) in a Greiner 96 well white plate. Leaf discs were washed once with sterile deionized (DI) water and then triggered with 1 μM flg22 and luminescence was measured for 30 min (Tristar^2^ LB 942; Berthold Technologies, Bad Wilbad, Germany). The data was replicated three times in independent trials.

### Bacterial growth assay

Bacterial growth assays were performed as described previously (Kadota *et al*., 2014), with slight modification. We assessed the significance of CML41 activity in overall plant resistance by first infiltrating leaves of different *Arabidopsis* lines with the virulent bacterial pathogen *P. syringae* pv. *tomato* (*Pst*) DC3000 (OD = 0.0002). We next performed infections by surface inoculation with the less virulent, coronatine deficient strain DC3000 (*cor* ^−^). Briefly, *Pst* bacterial suspension with OD_600 nm_ = 0.2 in 0.02% Silwet L‐77 were generously sprayed onto leaf abaxial and adaxial surfaces of 5–6‐wk‐old plants. Plants were covered during the course of infection and leaf discs were taken 3 h post‐inoculation (day 0) or 3 d post‐inoculation (day 3) from three leaves per plant, with six plants per genotype per independent trial. Bacterial growth was assessed by colony counting.

### GFP bombardment assay

Microprojectile bombardment assays were performed as previously described (Faulkner *et al*., 2013). Bombardment sites were assessed by epifluorescence microscopy (Leica DM6000).

## Results and Discussion

**Figure 1:**
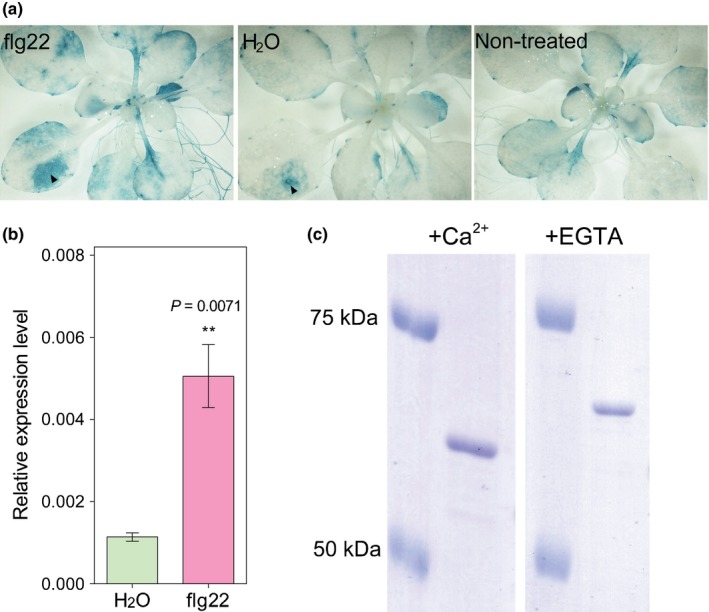
*CML41* is induced by flg22 in leaves and binds Ca^2+^. (a) GUS histochemical staining of 24‐d‐old *proCML41*::*GUS* plants treated with either H_2_O or flg22 infiltration for 4 h, as well as nontreatment control as indicated. Both flg22 and H_2_O injection at the wound/infiltration site is indicated by arrowheads, there was a localized increase in GUS activity induced by both flg22 and H_2_O injection at the wound site. (b) Quantitative RT‐PCR analysis of *CML41* in the leaves of 5–6‐wk‐old wildtype *Arabidopsis* Col‐0 plants grown in short‐day conditions (with 9 h : 15 h, light : dark) pre‐infiltrated with either H_2_O (green) or 1 μM flg22 (magenta) for 12 h. Gene transcript level was relative to *GAPDH‐A* (At3g26650). Data represent the mean ± standard error of the mean (SEM),* n *=* *3 biological replicates. Primer pairs used for (b) listed in Supporting Information Table S1. Statistical difference was determined by Student's *t‐*test, asterisks indicate statistical significance, *P*‐value as indicated. (c) Gel shift Ca^2+^ binding assay, purified recombinant MBP‐CML41 protein was separated on 8% SDS‐PAGE gel in the presence of 1 mM CaCl_2_ or 10 mM EGTA, the mobility of proteins was determined by comparison with the Precision Plus Protein™ Standards as indicated.

The 50 members of the *CML* gene family of *A. thaliana* have been proposed to encode proteins that facilitate specificity during Ca^2+^ signalling (McCormack *et al*., 2005; Bender & Snedden, 2013). *CML41* expression was previously shown to be upregulated by flg22 (Denoux *et al*., 2008), so we investigated whether it had a role in pathogen responses. First, we confirmed that *CML41* transcription was induced flg22 using quantitative RT‐PCR and expression analysis of the *CML41* promoter (Fig. 1a,b). We next investigated whether CML41 could bind Ca^2+^ and, therefore, has the potential to decode flg22‐induced Ca^2+^‐signals. CMLs can change conformation upon Ca^2+^ binding (Bender & Snedden, 2013), to test whether this is the case for CML41 we performed an electrophoresis mobility shift assay (Garrigos *et al*., 1991). Initial attempts to express and purify CML41 in *E. coli* were unsuccessful as the purified CML41 was insoluble. Therefore, CML41 was tagged with MBP to enhance its solubility and probed by western blot (Kapust & Waugh, 1999) (Fig. S1). The purified and soluble MBP‐CML41 fraction migrated faster in the presence of Ca^2+^ relative to EGTA (Fig. 1c), indicating its ability to bind Ca^2+^. This result is consistent with the recent report that CML41 binds to phenyl sepharose in a Ca^2+^‐dependent manner (Dell'Aglio *et al*., 2016). Attempts to obtain the Ca^2+^‐binding affinity for CML41 using microscale thermophoresis in a range of buffers were unsuccessful due to protein aggregation, even when tagged with MBP. However, the presence in CML41 of EF‐hands, which are conserved in other CML and have already been shown to bind Ca^2+^ in the nanomolar range, suggests that the Ca^2+^ responsiveness of CML41 could have a physiological role (Fig. S2).

**Figure 2:**
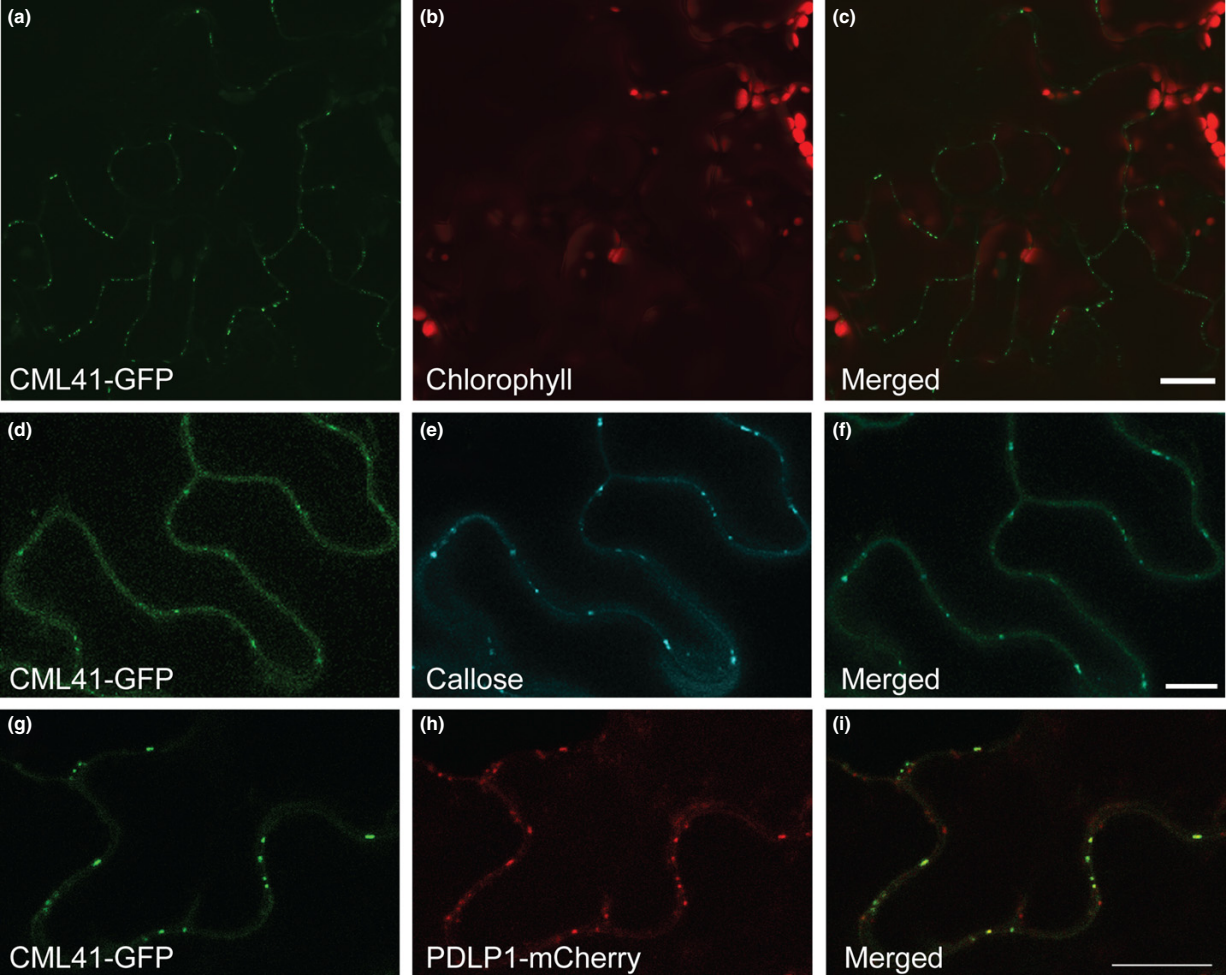
CML41 localizes to plasmodesmata. (a–c) Confocal image of CML41 tagged with GFP in the leaves of 5–6‐wk‐old *35S::CML41‐GFP Arabidopsis* plant; bars, 20 μm. (d–f) Co‐localization of CML41‐GFP (d) with callose stained by aniline blue (e) in the leaves of 5–6‐wk‐old *35S::CML41‐GFP* Arabidopsis leaf; bars, 10 μm. (g–i) Co‐localization of CML41‐GFP (g) with PDLP1‐mCherry (h), transiently expressed in *Nicotiana benthamiana*; bars, 10 μm.

To investigate the subcellular localization of CML41 we overexpressed *CML41* fused with *GFP* (*CML41‐GFP*). In leaves, CML41‐GFP localizes to punctate spots at the cell periphery (Fig. 2a–c), patterning that is reminiscent of plasmodesmata (Thomas *et al*., 2008; Simpson *et al*., 2009). We confirmed CML41‐GFP was localized to plasmodesmata by co‐staining plasmodesmal callose in Arabidopsis with aniline blue (Fig. 2d–f) or with Plasmodesmata‐located Protein 1 (PDLP1) in *Nicotiana benthamiana* (Fig. 2g–i) (Thomas *et al*., 2008). Plasmodesmal‐association has not yet been observed for any other CMLs, suggesting a novel role for CML41 of decoding flg22‐induced Ca^2+^ signals at plasmodesmata (Bender & Snedden, 2013).

**Figure 3:**
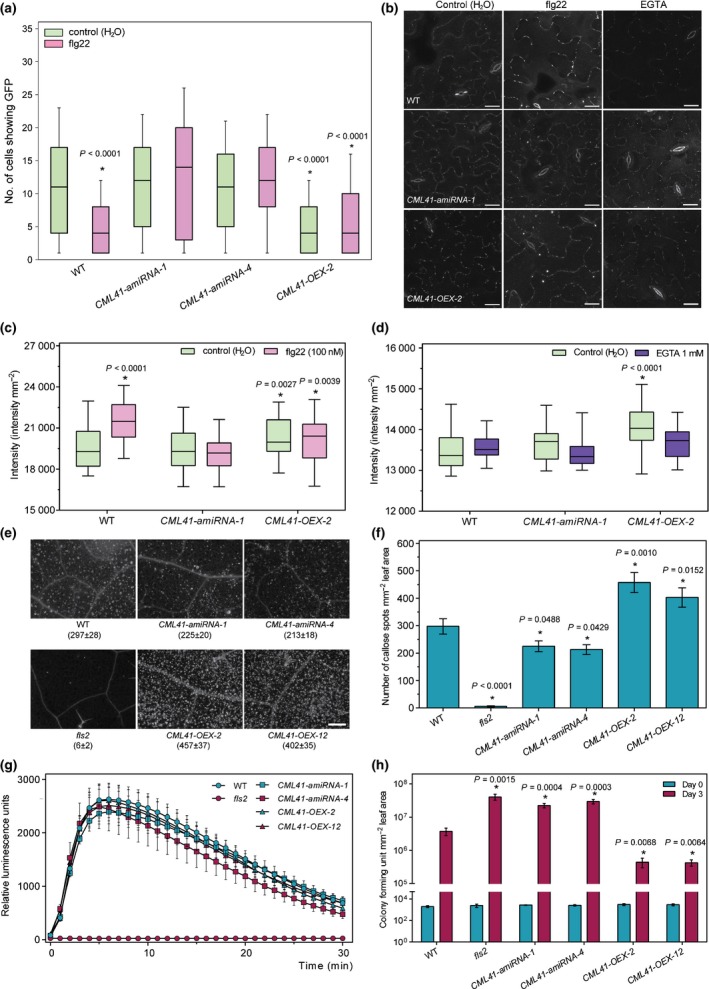
CML41 negatively modulates plasmodesmatal permeability and positively regulates callose production and plant defence. (a) Plasmodesmata permeability of wildtype (WT) Arabidopsis and *CML41* transgenic lines (*CML41‐amiRNA‐1*,* ‐4* and *CML41‐OEX‐2*) in response to 100 nM flg22. Plants were bombarded with constructs capable of producing GFP. Diffusion of GFP to surrounding cells provided a measure of molecular flux through plasmodesmata. Plants were infiltrated with flg22 2 h after bombardment. In each box‐plot, the white line indicates the median value, the shaded area represents the lower and upper quartiles, and the error bars indicate the minimum and maximum values, *n *=* *187 cells for WT (control), 137 for WT (flg22), 85 for *CML41‐amiRNA‐1* (control), 103 for *CML41‐amiRNA‐1* (flg22), 145 for *CML41‐amiRNA‐4* (control), 163 for *CML41‐amiRNA‐1* (flg22), 666 for *CML41‐OEX‐2* (control) and 198 for *CML41‐OEX‐2* (flg22). Statistical difference from the WT control was determined by Student's *t*‐test, asterisks indicate statistical significance, *P*‐values as indicated. (b) Confocal images of aniline blue stained plasmodesmal callose in the leaves of 5–6‐wk‐old Arabidopsis WT, *CML41‐OEX‐2* and *CML41‐amiRNA*‐1 lines upon H_2_O, flg22 and EGTA treatment for 30 min; bars, 20 μm. (c, d) Quantification of PD callose fluorescence intensity in the leaves of 5–6‐wk‐old Arabidopsis WT, *CML41‐OEX‐2* and *CML41‐amiRNA*‐1 lines following flg22 (c) and EGTA treatment (d). Box plots in (c) and (d) are as mentioned earlier, *n *=* *24 (c) and 27 (d). Statistical difference from the WT control was determined by Student's *t*‐test, asterisks indicate statistical significance, *P*‐values as indicated. (e) Macroscopy images and (f) quantification of callose deposition upon flg22 or H_2_O treatments in WT Col‐0, *fls2*,*CML41‐amiRNA‐1*,* ‐4* and *CML41‐OEX‐2*,* ‐12* lines upon 1 μM flg22 for 24 h, as indicated; bars, 200 μm in (e). Data represent the mean ± SEM,* n *=* *18 leaves. Statistical difference as determined by one‐way analysis of variance (ANOVA), asterisks indicate statistical significance, *P*‐values as indicated. (g) Reactive oxygen species (ROS) production stimulated by 1 μM flg22 was monitored in Arabidopsis WT, *fls2*,*CML41‐amiRNA‐1*,* ‐4* and *CML41‐OEX‐2*,* ‐12* leaf discs recorded at every minute using a luminol assay in a microplate reader, *fls2* was used as a control. Data are given as relative luminescence units and represent in mean ± SEM, from three independent trials with six technical replicates per biological replicate, *n *=* *6. (h) Evaluation of *CML41* transgenic plant susceptability to *Pst* DC3000 *cor* ^−^; quantification of bacterial growth in *fls2*,*CML41‐amiRNA‐1*,* ‐4*,*CML41‐OEX‐2*,* ‐12* lines and Arabidopsis WT plants upon 0 and 3 d post‐inoculation of *Pst* DC3000 *cor* ^−^ suspension. The bacterial colony number was counted in colony‐forming unit (CFU) per cm^2^. Data represent mean ± SEM,* n *=* *6. Statistical difference as determined by multiple Student's *t*‐test, asterisks indicate statistical significance from WTcontrol or flg22 treated, *P*‐values as indicated. The experiments were repeated three times with similar results. See Supporting Information Fig. S7 for the equivalent assays using *Pst* DC3000.

To further investigate the role of CML41 at plasmodesmata, we generated *CML41* gain‐ and loss‐of‐function transgenic lines using either gene overexpression (OEX) or artificial micro RNA (amiRNA) as no T‐DNA lines were available (Fig. S3). To determine whether CML41 has a role in flg22‐induced plasmodesmal closure we performed intercellular flux assays by measuring GFP diffusion from single cell transformation sites (Faulkner *et al*., 2013). Whilst flg22 treatment reduced spread of GFP in wildtype plants, indicating plasmodesmal closure, *CML41‐amiRNA* lines did not close their plasmodesmata in response to flg22 (Fig. 3a). *CML41‐OEX* plants showed increased basal levels of plasmodesmal closure (reduced spread of GFP), which was unaffected by flg22‐treatment (Fig. 3a).

To test whether CML41‐mediated, flg22‐induced plasmodesmal closure is executed by apoplastic callose deposition adjacent to plasmodesmata (Maule *et al*., 2012), we used automated image analysis to quantify plasmodesmata‐located aniline blue fluorescence. At 30 min post‐treatment, plasmodesmal callose was significantly increased in wildtype plants (Fig. 3b,c), supporting a model where flg22 induces plasmodesmal closure via rapid callose deposition. *CML41‐amiRNA* lines showed no difference in callose deposition between H_2_O and flg22 treated tissue (Fig. 3b,c), which coincided with the loss of the plasmodesmal closure response in these plants (Fig. 3a). *CML41*‐*OEX* plants had an increased presence of plasmodesmal callose in the basal state, which was reduced by the Ca^2+^‐chelator EGTA (Fig. 3b,d). These data indicate that Ca^2+^‐responsiveness of CML41 is likely to play a critical and physiologically relevant role in flg22‐induced plasmodesmal closure. Furthermore, this result implies that infiltration *per se* without EGTA (as performed in control experiments in Fig. 3a–f) may be sufficient to raise cytosolic Ca^2+^ concentration, as could be expected following a manipulation of the leaf apoplastic environment and water relations.

To examine whether CML41 is involved more broadly in pathogen‐associated molecular pattern (PAMP) responses we examined GFP movement in response to chitin, a fungal PAMP that reduces flux via plasmodesmata (Faulkner *et al*., 2013). *CML41‐amiRNA* plants showed the wildtype response to chitin (Fig. S4a); furthermore, chitin did not induce *CML41* transcription (Fig. S4b). This confirms that plants regulate plasmodesmal closure through different mechanisms following bacterial or fungal infection.

To assess CML41 specificity to plasmodesmal function we examined other flg22‐induced responses. Macroscopic callose deposition in the apoplast in response to flg22 was detectable 24 h post‐treatment (Gómez‐Gómez *et al*., 1999). In wildtype plants we observed widespread and greater callose deposition following flg22 infiltration compared to water treatment (Figs 3e,f, S5) and the *fls2* control (Gómez‐Gómez *et al*., 1999). The *CML41‐amiRNA* lines accumulated fewer callose deposits than wildtype plants after flg22 treatment (Fig. 3e,f). *CML41*‐*OEX* lines produced more callose deposits in response to flg22 (Fig. 3e,f).

Reactive oxygen species (ROS) accumulate rapidly upon flg22 treatment (Gómez‐Gómez *et al*., 1999; Boudsocq *et al*., 2010). While flg22‐induced ROS production is abolished in *fls2* (Gómez‐Gómez *et al*., 1999; Boudsocq *et al*., 2010), it was observed at similar levels to wildtype in *CML41*‐*amiRNA* and *CML41‐OEX* plants (Fig. 3g) indicating that CML41 does not play a role in flg22‐induced ROS production. The flg22 induces the expression of defence genes such as *flg22‐induced Receptor Kinase 1* (*FRK1*), *cytochrome P450 monooxygenase* (*CYP81F2*) and *NDR1/HIN1‐like 10* (*NHL10*) (Boudsocq *et al*., 2010). All these genes were significantly up‐regulated across all flg22‐infiltrated plants (Fig. S6). Transcript abundance of the salicylic acid (SA) inducible gene, *PATHOGENESIS‐RELATED 1* (*PR1*) (Wildermuth *et al*., 2001; Zipfel *et al*., 2004) is also enhanced by flg22 and showed no obvious difference between *CML41* transgenic lines and wildtype plants within the same treatments (Fig. S6). This contrasts the action of CML9 in the nucleus, which appears to negatively regulate *PR1* expression and the deposition of callose during flg22 responses (Leba *et al*., 2012). As the same transcriptional responses of these known early innate immunity induced genes are activated in wildtype, *CML41* overexpression and knockdown plants, this is strong evidence that CML41 does not work upstream of any of the corresponding response pathways.

In combination, this data establishes that CML41 functions in a specialized signalling pathway, downstream of FLS2 recognition of flg22. The localization of CML41 at plasmodesmata suggests that this signalling pathway directly regulates plasmodesmal function, closing the plasmodesmata within 30 min of pathogen perception (Fig. 3a). CML41 also functions in the production of large deposits of callose over a longer response timescale (Fig. 3e). It is not clear yet whether CML41 directly stimulates deposition via regulation of a callose synthase or inhibits a constitutive process of removal by interfering with β‐1,3‐glucanase activity (Luna *et al*., 2011), this may become clearer when interacting partners are identified for CML41. It should be noted that we detected *CML41* expression in the roots following flg22 treatment (Fig. 1a); therefore, it is likely that CML41 plays a similar role in PD flux regulation when challenged with soil bacterial pathogens. An investigation is also warranted into: the role of CML41 in the other tissues in which it is expressed such as senescent leaves and flowers (McCormack *et al*., 2005); and, the significance of its transcriptional regulation by RNA‐dependent methylation (Baev *et al*., 2010).

In this study we focused on assessing the significance of CML41 activity in overall plant resistance by surface inoculation of different Arabidopsis lines with *P. syringae* (Figs 3h, S6). Three days post‐infection (3 dpi), *CML41*‐*amiRNA* lines showed more bacterial growth than wildtype, similar to *fls2*, indicating they were more susceptible; while the *CML41‐OEX* lines showed less bacterial growth than wildtype (Fig. 3h). This suggests that flg22‐induced Ca^2+^ signalling via CML41 is a critical component of callose deposition. A parallel role for Ca^2+^ in a callose‐independent plasmodesmal closure pathway has also been proposed (Sager & Lee, 2014); however, this does not appear to play a major role in the conditions assayed here.

We have identified CML41 as a Ca^2+^ responsive component of defensive plasmodesmal closure. Chitin‐induced plasmodesmal closure has previously been shown to be critical to defence and overall resistance against a fungal pathogen and the data presented here identifies that flg22‐induced plasmodesmal closure is similarly critical to defence against bacterial pathogens. Beyond establishing new understanding of the mechanisms of plasmodesmal function via Ca^2+^, this highlights a role for symplastic connectivity in the full and complete execution of defence responses.

## Author contributions

M.G., C.F. and K.S. supervised the research. B.X. performed the majority of experiments except the following. B.H. cloned the amiRNA. B.X. and D.C. purified the protein and performed Ca^2+^ shift experiments. A.L. performed callose deposition, ROS burst and bacterial growth assays. C.C. performed bombardment assays and plasmodesmal callose quantification. T.S.G.O. wrote the image analysis script. C.C. and C.F. performed co‐localization experiments. B.X., C.C., A.L., K.S., M.G. and C.F. drafted the manuscript. All authors commented on the manuscript.

## Supporting information

Please note: Wiley Blackwell are not responsible for the content or functionality of any Supporting Information supplied by the authors. Any queries (other than missing material) should be directed to the *New Phytologist* Central Office.

**Fig. S1** CML41 was produced through heterologous expression in *Escherichia coli*.
**Fig. S2** *In silico* analysis of CML41 protein sequence and EF domains.
**Fig. S3** *CML41* transcript abundance was modified in transgenic *CML41* misexpression lines.
**Fig. S4** CML41 does not have a role in chitin response of plasmodesmata.
**Fig. S5** Callose production is not induced by water infiltration in CML41 overexpression or knockdown lines.
**Fig. S6** Early immune response gene transcription is not affected by CML41 overexpression or knockdown.
**Fig. S7** *CML41* overexpression increases bacterial resistance.
**Table S1** Primers used for quantitative RT‐PCR analysis and molecular cloning
