# Supplementary material for: A calmodulin‐like protein regulates plasmodesmal closure during bacterial immune responses
Source: New Phytol. 2017 May 17;215(1):77–84. doi: 10.1111/nph.14599 (PMC5488192; doi:10.1111/nph.14599)
Supplement: Supplementary file 1 — Fig. S1 CML41 was produced through heterologous expression in Escherichia coli. Fig. S2 In silico analysis of CML41 protein sequence and EF domains. Fig. S3 CML41 transcript abundance was modified in transgenic CML41 misexpression lines. Fig. S4 CML41 does not have a role in chitin response of plasmodesmata. Fig. S5 Callose production is not induced by water infiltration in CML41 overexpression or knockdown lines. Fig. S6 Early immune response gene transcription is not affected by CML41 overexpression or knockdown. Fig. S7 CML41 overexpression increases bacterial resistance. Table S1 Primers used for quantitative RT‐PCR analysis and molecular cloning [file NPH-215-77-s001.pdf]

Supporting Information

Title: A calmodulin-like protein regulates plasmodesmal closure during bacterial immune responses

Authors: Bo Xu, Cecilia Cheval, Anuphon Laohavisit, Bradleigh Hocking, David Chiasson, Tjelvar S G Olsson, Ken Shirasu, Christine Faulkner, Matthew Gilliham

Article acceptance date: 10 April 2017

The following Supporting Information is available for this article:

Table S1. Primers used for qRT-PCR analysis and molecular cloning.

Fig. S1. CML41 was produced through heterologous expression in *E. coli*.

Fig S2. *In silico* analysis of CML41 protein sequence and EF domains.

Fig. S3. *CML41* transcript abundance was modified in transgenic *CML41* misexpression lines.

Fig S4. CML41 does not have a role in chitin response of plasmodesmata.

Fig S5. Callose production is not induced by water infiltration in CML41 overexpression or knockdown lines.

Fig S6. Early immune response gene transcription is not affected by CML41 overexpression or knockdown.

Fig S7. *CML41* overexpression increases bacterial resistance.

Table 1. Primers used in this study for qPCR analysis and cloning. Upper case letters in CML41_amiRNA cloning primers indicate sequence that was mutated during PCR amplification from the pRS300 vector as a template, lower case letters in the CML41_amiRNA primer indicate sequence that binds to the pRS300 vector as a template and primes the PCR amplification.

Gene name	Primers	Sequence (5'-3')	Technique
UBI10	UBI10_qPCR_F1	CACACTCCACTTGGTCTTGCGT	qRT-PCR
	UBI10_qPCR_R1	TGGTCTTTCCGGTGAGAGTCTTCA	
GAPDH-A	GAPDH-A_qPCR_F1	GAGGACTTTGTTGGATTGATG	
	GAPDH-A_qPCR_R1	ATGGCTTCACACTCTCCGTA	
FRK1	FRK1_qPCR_F1	ATCTTCGCTTGGAGCTTCTC	
	FRK1_qPCR_R1	TGCAGCGCAAGGACTAGAG	
CYP81F2	CYP81F2_qPCR_F1	AATGGAGAGAGCAACACAATG	
	CYP81F2_qPCR_R1	ATACTGAGCATGAGCCCTTTG	
NHL10	NHL10_qPCR_F1	TTCCTGTCCGTAACCCAAAC	
	NHL10_qPCR_R1	CCCTCGTAGTAGGCATGAGC	
PR1	PR1_qPCR_F2	GCTCTTGTAGGTGCTCTTGTTCT	
	PR1_qPCR_R2	TGCCTCTTAGTTGTTCTGCGT	
CML41	CML41_qPCR_F1	AGCGACGGCGACGGTAAG	
	CML41_qPCR_R1	GTCAGTGTCAACTTCGTTTATCG	
	CML41_CDS_F	ATGGCAACTCAAAAAGAGAAACCT	cloning
	CML41_CDS_R	CTAAACCGTCATCATTTGACGAAAC	
CML41-stop	CML41_CDS_F_CACC	CACCATGGCAACTCAAAAAGAGAAACCT	
	CML41_CDS_R	CTAAACCGTCATCATTTGACGAAAC	
CML41-amiRNA	amiRNA_A	CTGCAAGGCGATTAAGTTGGGTAAC	
	CML41_amiRNA_IV	gaAAAACCGACATCATTTGATCActacatatatattccta	
	CML41_amiRNA_III	agTGATCAAATGATGTCGGTTTTtcacaggtcgtgatatg	
	CML41_amiRNA_II	agTGGTCAAATGATGACGGTTTAtcaaagagaatcaatga	
	CML41_amiRNA_I	gaTAAACCGTCATCATTTGACCActctcttttgtattcca	
	amiRNA_B	GCGGATAACAATTTCACACAGGAAACAG	
proCML41	proCML41_2kb_F1	TAACGCGCAAGGAAACGC	
	proCML41_2kb_R1	ATCATTGAGTTTATATGCTGTAGTGTGTT	

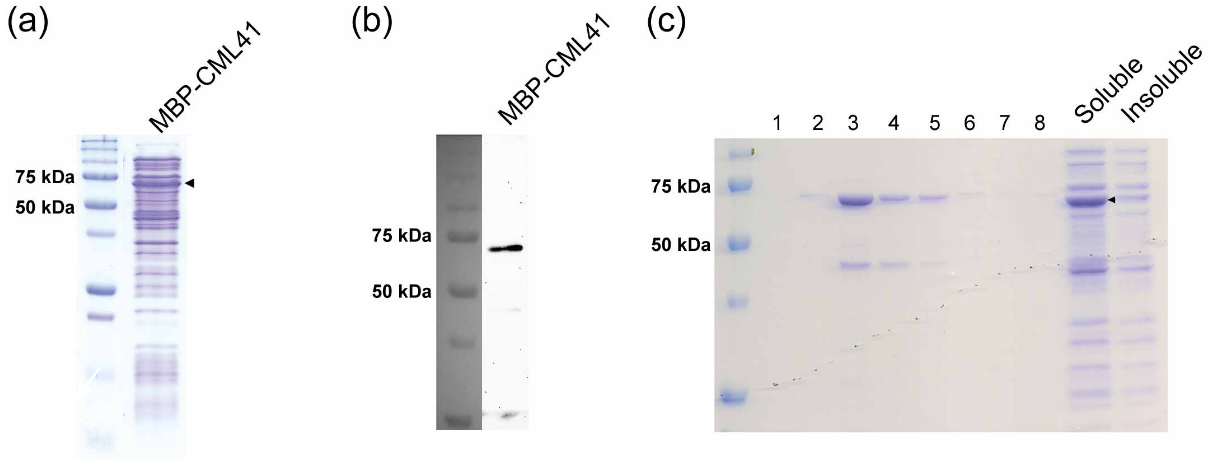

Fig S1. CML41 was produced through heterologous expression in *E. coli*. Protein expression, purification and western blot of CML41 tagged with Maltose Binding protein (MBP). **(a)** SDS-PAGE gel analysis of total soluble proteins extracted from *E. coli* expressing *CML41* tagged with *MBP*. **(b)** Further confirmation of soluble MBP-CML41 by Western blot on total soluble proteins extracted, against anti-MBP monoclonal antibody (New England Biolabs). **(c)** SDS-PAGE gel analysis of fractions 1-8 obtained after elution from Poly-Prep® Chromatography Columns (Bio-Rad), total soluble and insoluble proteins as indicated. Protein size was compared with the Precision Plus Protein™ Standards (Bio Rad). Arrows point to soluble MBP-CML41 protein in **(a,c)**.

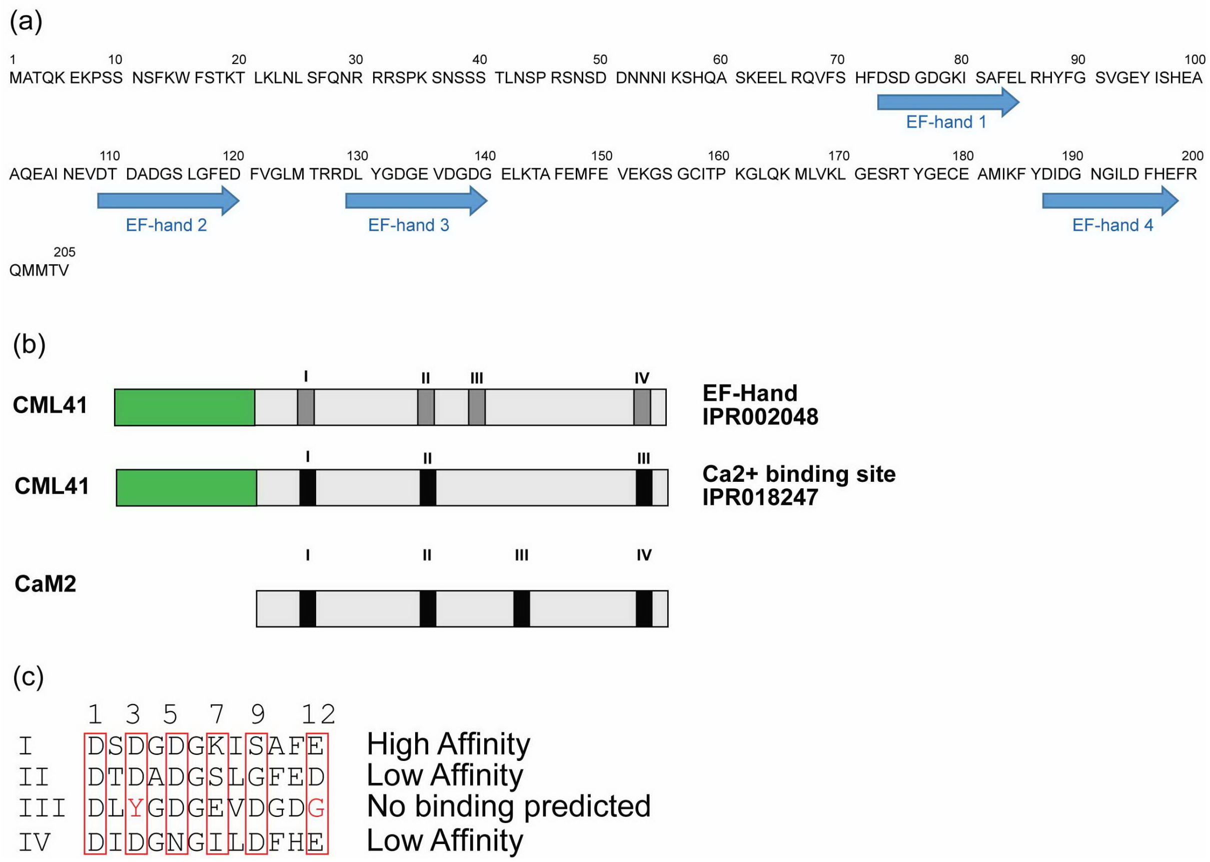

Fig S2. *In silico* analysis of CML41 protein sequence and EF domains. (a) CML41 protein sequence and predicted EF hand domains, as indicated by arrows. **(b)** Predicted Ca^{2+}-binding EF-hand sites of CML41, it contains an N-terminal extension (green) relative to CaM2 (representative conserved CaM) and three predicted Ca^{2+}- binding EF hands (black bars). **(c)** Amino acid sequence of each EF hand and the predicted binding affinity from Cal-EF-AFi (http://202.41.10.46/calb/index.html). Red boxes correspond to predicted Ca^{2+} co-ordinating residues; non-conforming residues are shown in red text. Please note that the equivalent EF hand 1 in CML42 has been shown to have a K_d for Ca^{2+} in the low nanomolar range, and III and IV are predicted to have an affinity in the low micromolar range, data extracted from the Ca^{2+} binding CML42 and CML43 (Dobney *et al.*, 2009; Bender *et al.*, 2014).

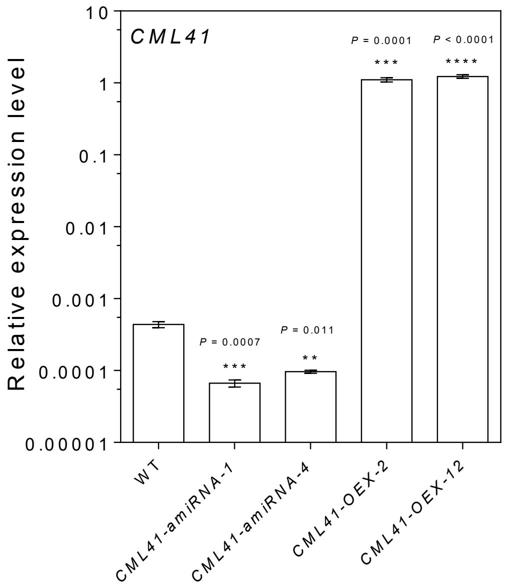

Fig. S3. *CML41* transcript abundance was modified in transgenic *CML41* misexpression lines. *CML41* transcript level in 5-6 week-old wildtype and *CML41* transgenic plants (*CML41-amiRNA-1*, *-4* and *CML41*-OEX-*2*, *-12* lines). The overexpression of *CML41*-amiRNA reduced expression of *CML41* by up to 84% in the lines *CML41-amiRNA-1* and *-4*, *CML41* transcript level was dramatically increased by more than 2500- and 4000-fold respectively in overexpression lines *CML41*-OEX-*2* and *-12* in comparison to control wildtype Col-0 plantsin standard conditions. Data are the mean ± SEM, n = 5 biological replicates. Primer pairs used as listed in Table S1. Gene transcript level was relative to *GAPDH-A* (At3g26650). Statistical difference from wildtype was determined by *Student's* t-test, asterisks indicate statistical, *P* values as indicated.

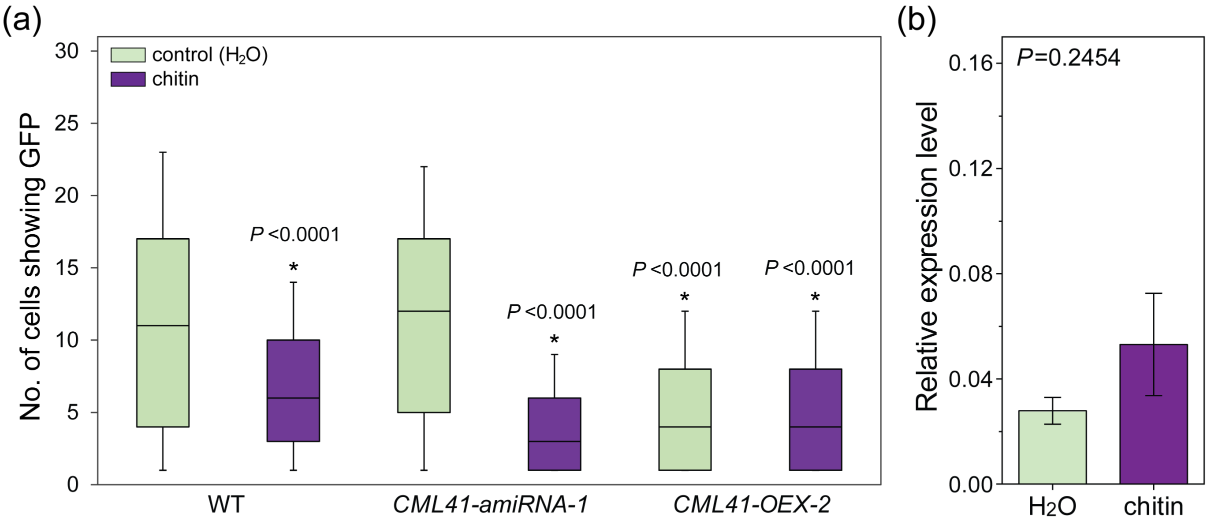

Fig. S4. **CML41 does not have a role in chitin response of plasmodesmata. (a)** diffusion of GFP to surrounding cells provides a measure of molecular flux through plasmodesmata following chitin treatment. Plants were infiltrated with H_2O or chitin (0.5 mg/ml) 2 hrs after bombardment. In each box-plot, the white line indicates the median value, the shaded area represents the lower and upper quartiles, and the error bars indicate the minimum and maximum values, n = 187 cells for WT (control), 224 for WT (chitin), 137 for *CML41-amiRNA-1*, 317 for *CML41-amiRNA-1* (chitin), 503 for *CML41-OEX*-2 (control) and 164 for *CML41-OEX-2* (chitin). **(b)** qRT-PCR analysis of *CML41* expression of 5-6 week-old wildtype (Col-0) *Arabidopsis* plants in either H_2O control (green) or 12 h after (0.5mg/ml) chitin (magenta). Gene transcript level was relative to *UBI10* (At4g05320). Data represent the mean ± SEM, n = 4 biological replicates. Primer pairs used for **(b)** listed in Table S1. Statistical difference from wildtype control samples was determined by *Student's* t-test in **(a,b)**, asterisks indicate statistical, *P* values as indicated.

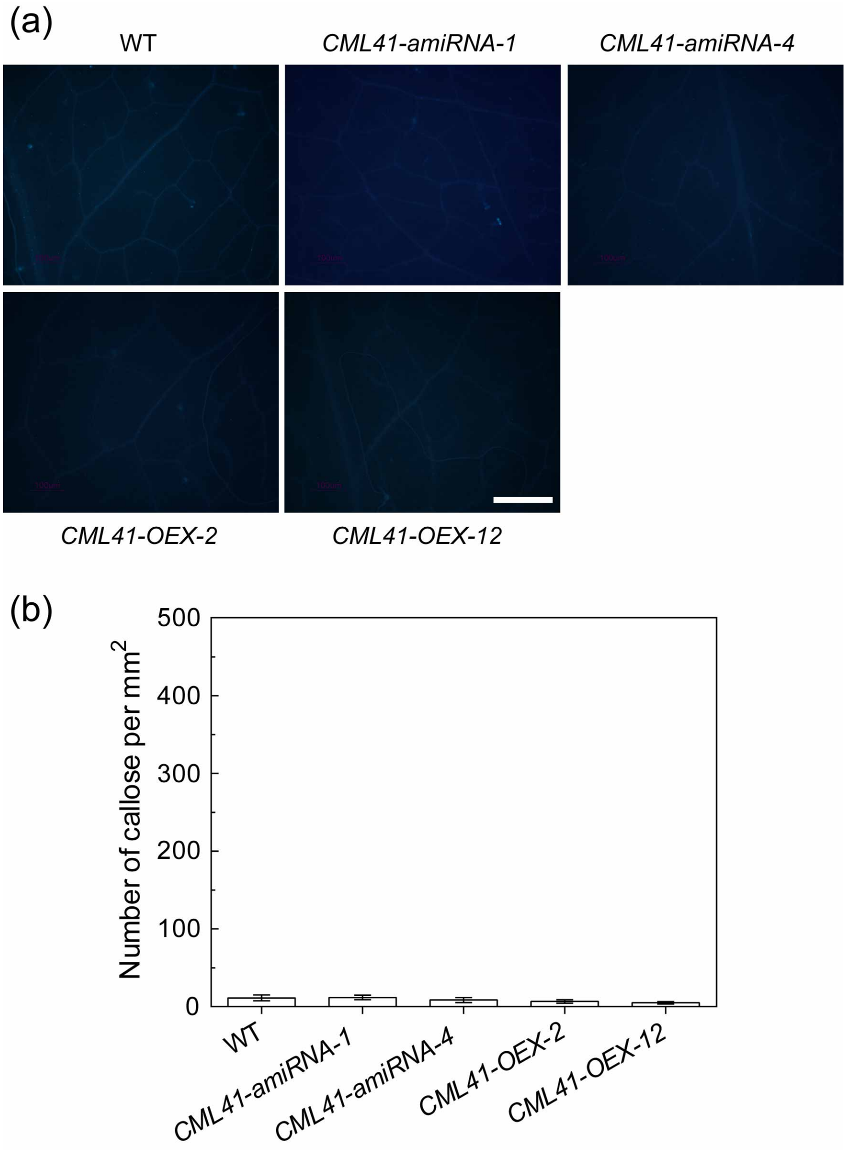

Fig. S5. Callose production is not induced by water infiltration in CML41 overexpression or knockdown lines. (a) callose production in the leaves of in 5-6 week-old wildtype and *CML41* transgenic plants (*CML41-amiRNA-1*, *-4* and *CML41*-OEX-*2*, *-12* lines) following H_2O treatment. **(b)** no obvious callose was produced in the leaves of *CML41*-OEX-*2, -12*, *CML41-amiRNA-1*, *-4* and wildtype plants upon H_2O pre-infiltration for 24 h, n = 9, scale bars = 100 μm.

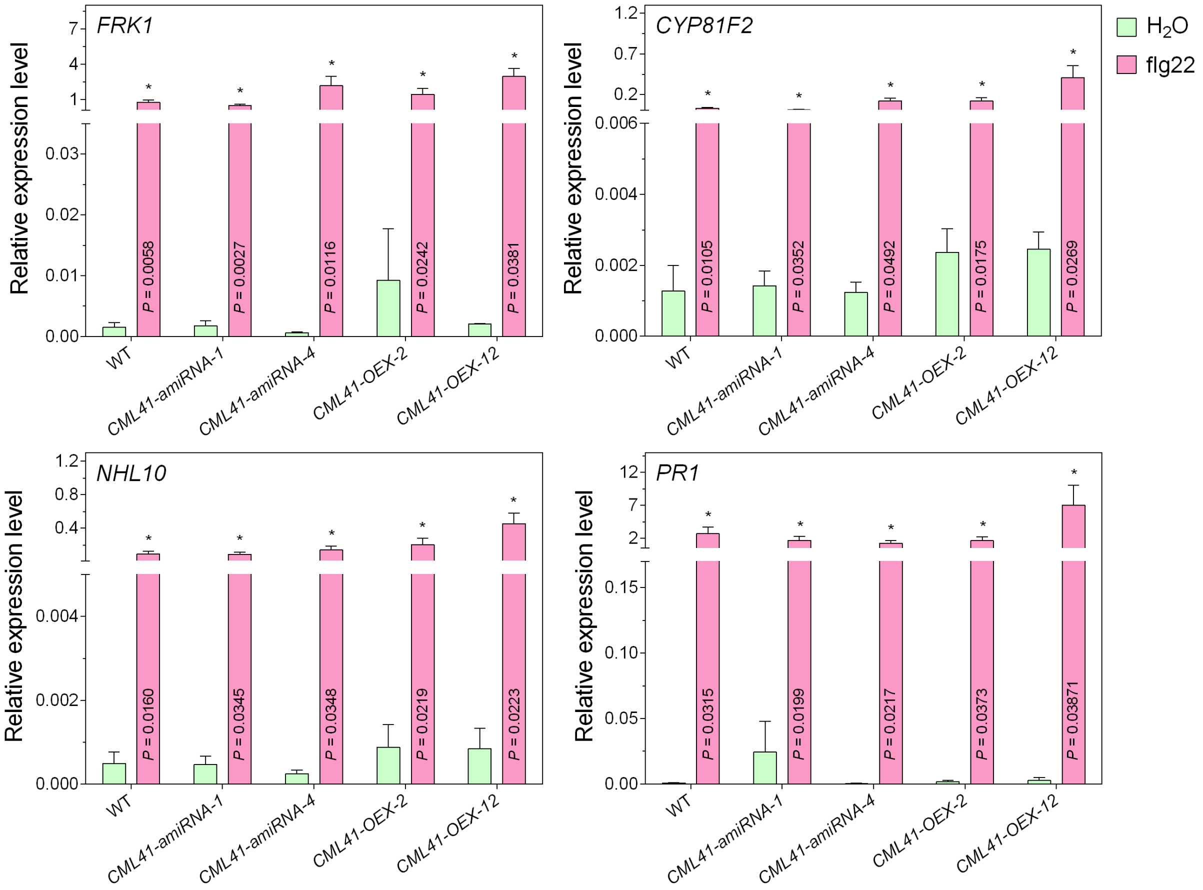

Fig. S6. Early immune response gene transcription is not affected by CML41 overexpression or knockdown. *FRK1, CPY81F2, NHL10* and *PR1* transcript level in the leaves of in wildtype and *CML41* transgenic plants (*CML41-amiRNA-1*, *-4* and *CML41*-OEX-*2*, *-12* lines) following either flg22 or H_2O treatment. Leaves of 5-6 week-old plants were infiltrated with either 2 µM flg22 or H_2O for 12 h before harvest. Primer pairs used as listed in Table S1, data represent in mean ± SEM, n = 3 biological replicates of wildtype plants with flg22 treatments, *CML41-amiRNA-1* with flg22 treatments and *CML41-OEX-12* with H_2O and flg22 treatments; n = 4 biological replicates of the rest of samples. Gene transcript level was relative to *GAPDH* (At3g26650). Statistical difference as determined by *Student's* t-test, asterisks indicate statistical significance, *P* values as indicated.

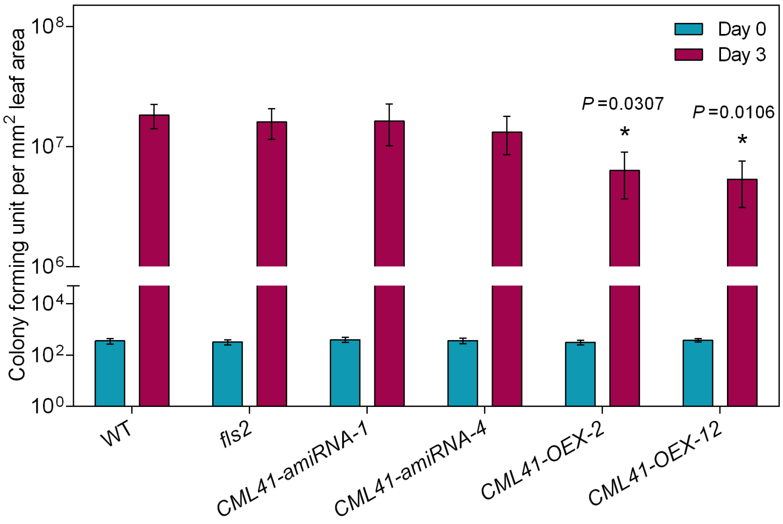

Fig. S7. *CML41* overexpression increases bacterial resistance. Bacterial pathogenesis assays on transgenic plants misexpressing CML41 using *Pto* DC3000. Quantification of bacterial growth in *fls2*, *CML41-amiRNA-1*, *-4*, *CML41*-OEX-2, *-12* lines and wiltype plants 0 and 3 days post inoculation of *Pto* DC3000 suspension. The bacterial colony number was counted in colony-forming unit (c.f.u) per cm^2. Data are mean ± SEM, n = 6 biological replicates. Statistical difference as determined by Two-way ANOVA, asterisks indicate statistical significance, *P* values as indicated. The experiments were repeated at least three times with similar results. A difference was observed between *CML41-OEX* lines and wildtype plants as occurred using the cor^- strain (Fig 3h). Interestingly, unlike that observed in fig 3h using Pst DC3000 cor^-, when using Pst DC3000, the amiRNA lines showed no significant differences compared to WT over the time period assayed here. It is important to note that in these assays the *fls2* knockout plants (our positive control) also showed no increase in pathogen susceptibility over wildtype using Pst DC3000. In our hands these results are common and repeatable for many of the pathogen responsive genes. The reasons for this are not yet clear as there are many factors that may be involved in pathogen responses against DC3000 compared with the cor^- strain.

References

Bender KW, Dobney S, Ogunrinde A, Chiasson D, Mullen RT, Teresinski HJ, Singh P, Munro K, Smith SP, Snedden WA. 2014. The Calmodulin-like protein CML43 functions as a salicylic-acid-inducible root-specific Ca^{2+} sensor in *Arabidopsis*. *Biochemical Journal* **457**:127-36.

Dobney S, Chiasson D, Lam P, Smith SP, and Snedden WA. 2009. The Calmodulin-related calcium sensor CML42 plays a role in trichome branching. Journal of Biological Chemistry **284**,31647-31657.
